# Supplementary material for: Measuring recent effective gene flow among large populations in Pinus sylvestris: Local pollen shedding does not preclude substantial long-distance pollen immigration
Source: PLoS One. 2021 Aug 13;16(8):e0255776. doi: 10.1371/journal.pone.0255776 (PMC8362938; doi:10.1371/journal.pone.0255776)
Supplement: S1 Appendix — (DOCX) [file pone.0255776.s001.docx]

**S1 Appendix. PCR protocols.**

Two different multiplex polymerase chain reaction (PCR) mixes, following the same PCR conditions, were used for cpSSR amplification. Both mixes contained 10ng of genomic DNA, 2x Master Mix, and 2μM Mix Primers in a total volume of 10μL, according to Type-it Microsatellite PCR Kit instructions (Qiagen, Venlo, Netherlands). Used primers were modified with a 5’ fluorochrome HEX FAM TAM or TET (Eurofins MWG Operon, Ebersberg, Germany) end label. One mix contained Pt30204, Pt36480 and Pt87268 primers, and the other Pt15169, Pt26081 and Pt71936 primers. We amplified cpSSR with a Veriti 96-well thermal cycler (Applied Biosystems, Foster City, California, USA), using the following touchdown PCR program for both multiplex PCR mixes: initial denaturation at 95 ºC for 5 min, followed by 18 cycles of 30 s at 95 ºC, 1 min 30 s at 57 ºC, and 30 s at 72 ºC, and a final extension step at 60 ºC for 30 min.
