# Supplementary material for: Measuring recent effective gene flow among large populations in Pinus sylvestris: Local pollen shedding does not preclude substantial long-distance pollen immigration
Source: PLoS One. 2021 Aug 13;16(8):e0255776. doi: 10.1371/journal.pone.0255776 (PMC8362938; doi:10.1371/journal.pone.0255776)
Supplement: S2 Appendix — (DOCX) [file pone.0255776.s002.docx]

**S2 Appendix. Details of the MCMC algorithm.**

We approximated the posterior probability density of model parameters using the Metropolis-Hastings algorithm [1,2]. We employed two steps at each iteration of the algorithm, with either **m** or **p** being potentially modified at each step. Initial values for **p** were set at their posterior frequency, given the empirically observed haplotypic counts, under the prior assumption that all the populations have an identical set of *nh* equifrequent haplotypes [3]. The frequency of the *k-*th haplotype at the *j*-th population was thus initially set at ${\hat{\text{p}}}_{\text{jk}}\text{=}\left( \text{n}_{\text{jk}}\text{+1/}\text{nh} \right)\text{/}\left( \text{A}_{\text{j}}\text{+1} \right)$, where *n_jk_* is the empirically observed count of haplotype *k* at population *j*, *A_j_* is the empirical sample size for population *j*, and *nh* is the observed number of haplotypes across all populations. Initial values for **m** were set at their maximum-likelihood estimates, using eqn. 2 and fixed **p** at its initial values.

*Updating offspring proportions (***m***)*

We updated offspring proportions of all *I* recipient populations at every iteration, one population at a time. For each recipient population *i*, we randomly chose two different elements *q* and *r* of vector **m***_i_*, and propose a new value $m_{iq}^{'}$= *u*, where *u* ~ *U*[max(0, *m_iq_*− *e_m_*), min(*m_iq_*+ *e_m_*, *m_iq_*+ *m_ir_*)] and *e_m_* was some incremental value that was tuned to obtain reasonable acceptance rates. We then set $m_{ir}^{'}$= *m_iq_* + *m_ir_* −$m_{iq}^{'}$. The updated vector $\mathbf{m}^{'}$, constructed by replacing elements *m_iq_* and *m_ir_* of **m** with $m_{iq}^{'}$ and $m_{ir}^{'}$, was accepted with probability

$\alpha(\mathbf{m}^{'}|\mathbf{m})=\min\left(1, \frac{\Pr(\mathbf{n}|\mathbf{m}^{'},\mathbf{p})f(\mathbf{m}^{'})q(\mathbf{m},\mathbf{m}^{'})}{\Pr(\mathbf{n}|\mathbf{m},\mathbf{p})f(\mathbf{m})q(\mathbf{m}^{'},\mathbf{m})}\right)$,

where *q*(**m**, $\mathbf{m}^{'}$) is the uniform density described above and *q*($\mathbf{m}^{'}$, **m**) is the uniform density corresponding to the reverse move, namely *u* = *U*[max(0, $m_{iq}^{'}$− *e_m_*), min($m_{iq}^{'}$+ *e_m_*, $m_{iq}^{'}$+$m_{ir}^{'}$)].

*Updating adult population haplotypic frequencies (***p***)*

We updated adult haplotypic frequencies of all *J* candidate source populations at every iteration, one population at a time. For each source population *j*, we randomly chose two of the *K* haplotypes, *k* and *l*, and propose $p_{jk}^{'}$= *u*, where *u*~ *U*[max(0, *p_jk_* − *e_p_*), min(*p_jk_* + *e_p_*, *p_jk_* + *p_jl_*)] and *e_p_* is some incremental value that was adjusted to obtain reasonable acceptance rates. We then set $p_{jl}^{'}$= *p_jk_* + *p_jl_* −$p_{jk}^{'}$ and accepted the move with probability

$\alpha(\mathbf{p}^{'}|\mathbf{p})=\min\left(1, \frac{\Pr(\mathbf{n}|\mathbf{m},\mathbf{p}^{'})\Pr(\mathbf{N}|\mathbf{p}^{'})f(\mathbf{p}^{'})q(\mathbf{p},\mathbf{p}^{'})}{\Pr(\mathbf{n}|\mathbf{m},\mathbf{p})\Pr(\mathbf{N}|\mathbf{p})f(\mathbf{p})q(\mathbf{p}^{'},\mathbf{p})}\right)$,

where $\mathbf{p}^{'}$ is the vector **p** with elements *p_jk_* and *p_jl_* replaced with $p_{jk}^{'}$ and $p_{jl}^{'}$, *q*(**p**, **p’**) is the uniform density described above, and *q*(**p’**, **p**) is the reverse move uniform density *U* [max(0, $p_{jk}^{'}$ − *e_p_*), min($p_{jk}^{'}$ + *e_p_*, $p_{jk}^{'}$ + $p_{jl}^{'}$)].

*MCMC chain parameters*

Incremental values of the proposal distributions were adjusted in pilot runs to obtain acceptance rates between 40-60%. We then used a total of 260 000 MCMC iterations, discarding the first 10 000 as burn-in cycles and considering a thinning step of 25 iterations, which yields 10 000 posterior distribution samples.

**References**

1. Metropolis N, Rosenbluth AW, Rosenbluth MN, Teller AH, Teller E. Equation of state calculations by fast computing machines. J Chem Phys. 1953;21: 1087–1092. doi:http://dx.doi.org/10.1063/1.1699114

2. Hastings WK. Monte carlo sampling methods using Markov chains and their applications. Biometrika. 1970;57: 97–109. doi:10.1093/biomet/57.1.97

3. Rannala B, Mountain JL. Detecting immigration by using multilocus genotypes. Proc Natl Acad Sci U S A. 1997;94: 9197–9201. doi:10.1073/pnas.94.17.9197
