# Supplementary material for: Measuring recent effective gene flow among large populations in Pinus sylvestris: Local pollen shedding does not preclude substantial long-distance pollen immigration
Source: PLoS One. 2021 Aug 13;16(8):e0255776. doi: 10.1371/journal.pone.0255776 (PMC8362938; doi:10.1371/journal.pone.0255776)
Supplement: S3 Appendix — (DOCX) [file pone.0255776.s003.docx]

**S3 Appendix. Monte Carlo analysis of method performance.**

Given the actual numbers of successfully genotyped adult and offspring individuals in each population, namely **A** = {196, 198, 397} and **O** = {196, 188}, the assumed adult population haplotypic frequencies $\hat{\mathbf{p}}$ (see below and Table S1), and the assumed offspring proportions from different paternal origins **m** for each simulated scenario (Table 1), simulations involved three steps:

(1) For each source population *j*, we simulated an adult haplotypic sample by drawing *A_j_* times from a multinomial distribution with class probabilities $\hat{\mathbf{p}}_{j}$.

(2) For each recipient population *i*, we simulated an offspring sample in which the number of individuals sired by fathers from each source population was drawn from a multinomial distribution with three classes with probabilities {*m_i_*_1_, *m_i_*_2_, *m_i_*_3_} and *O_i_* trials. Next, the haplotype of each individual offspring sired by a father in population *j* was drawn from a multinomial distribution with class probabilities $\hat{\mathbf{p}}_{j}$.

(3) Given the simulated adult and offspring samples, we estimated posterior distributions of parameters using eqn. 4 and the MCMC algorithm described in Appendix S2.

Simulated adult population haplotypic frequencies ($\hat{\mathbf{p}}$) were set at their posterior frequency, given the empirically observed haplotypic counts, under the conservative prior assumption that all the populations have an identical set of *nh* equifrequent haplotypes [1]. Specifically, the frequency of the *k-*th haplotype at the *j*-th population was set at ${\hat{\text{p}}}_{\text{jk}}\text{=}\left( \text{n}_{\text{jk}}\text{+1/}\text{nh} \right)\text{/}\left( \text{A}_{\text{j}}\text{+1} \right)$, where *n_jk_* is the empirically observed count of haplotype *k* at population *j*, *A_j_* is the empirical sample size for population *j*, and *nh* is the assumed total number of haplotypes across all populations. To explore potential biases resulting from low-frequency haplotypes undetected in field samples, we considered three alternative values of *nh* in the simulations: either equal to, twice as large as, or four times as large as the total number of observed haplotypes across all empirical adult and offspring samples, namely *nh =* 247, 494 or 988 (see Table S1). Note that, once adult population frequencies have been set at their assumed values and used to generate adult and offspring samples (steps 1 and 2), they are subsequently considered to be unknown and are estimated from simulated samples during inference (step 3 above). The simulation scheme will reflect errors owing to small adult and offspring samples, including uncertainty in haplotype frequencies estimation.

For each simulated dispersal scenario (i.e. for each assumed **m**; Table 1) and assumed adult population haplotypic frequencies (Table S1), we conducted *R* = 1 000 independent replications of the three steps to obtain 1 000 $\hat{\mathbf{m}}$values, which represent the expected distribution of **m** estimates produced by our model under the empirical sample sizes and assumed adult population haplotypic frequencies. We also compared $\hat{\mathbf{m}}$ against **m** to calculate the expected bias, RMSE and the non-coverage rate of the 95% credibility interval (CI) of the estimates, distinguishing the proportion of times that the CI lower limit is larger than the assumed value (*LLTH*) and the proportion of times that the CI upper limit was lower than the assumed value (*ULTL*):

$$Bias(\hat{m}_{ij})=\frac{1}{R}\sum_{r=1}^{R} \left(\hat{m}_{ij}^{r}-m_{ij}\right)$$

$$RMSE(\hat{m}_{ij})=\left[\frac{1}{R}\sum_{r=1}^{R} \left(\hat{m}_{ij}^{r}-m_{ij}\right)^{2}\right]^{0.5}$$

$$LLTH\left[\mathrm{CI}(\hat{m}_{ij})\right]=\frac{1}{R}\sum_{r=1}^{R} Q_{L}\left[CI(\hat{m}_{ij}^{r}),m_{ij}\right]$$

$$ULTL\left[\mathrm{CI}(\hat{m}_{ij})\right]=\frac{1}{R}\sum_{r=1}^{R} Q_{U}\left[CI(\hat{m}_{ij}^{r}),m_{ij}\right]$$

where $\hat{m}_{ij}^{r}$ is the estimated (using the median of the posterior) proportion of offspring from the *i*-th population sired by fathers from the *j*-th population for the *r*-th replicate in the simulations, $CI(\hat{m}_{ij}^{r})$ is the 95% credibility interval of $\hat{m}_{ij}^{r}$ obtained from the 0.025 and 0.975 percentiles of the posterior distribution, and *Q_L_* (respectively *Q_U_*) is an indicator function with value 1 if the lower limit of $CI(\hat{m}_{ij}^{r})$is larger (respectively the upper limit lower) than *m_ij_* or zero otherwise

**References**

1. Rannala B, Mountain JL. Detecting immigration by using multilocus genotypes. Proc Natl Acad Sci U S A. 1997;94: 9197–9201. doi:10.1073/pnas.94.17.9197
