# Supplementary material for: Measuring recent effective gene flow among large populations in Pinus sylvestris: Local pollen shedding does not preclude substantial long-distance pollen immigration
Source: PLoS One. 2021 Aug 13;16(8):e0255776. doi: 10.1371/journal.pone.0255776 (PMC8362938; doi:10.1371/journal.pone.0255776)
Supplement: Attachment — Submitted filename: response_letter.pdf [file pone.0255776.s007.pdf]

REBUTAL LETTER [PONE-D-21-09948]

(**NOTE**: the line numbers referenced below refer to the marked-up copy of the manuscript that highlights changes made to the original version, and *not* to the unmarked revised version)

EDITOR COMMENTS

COMMENT: *The manuscript has been met with great interest on the side of the review team. Before acceptance, I would like to see a reply to the comments and many suggestions, as detailed further below. Overall, to me this is someway between a 'minor' and 'major' revision (the reviewers may want to see the revised version, out of their pure interest in the first place).*
The manuscript has been reviewed by three colleagues who seem to be very interested in the approach and the results. They have all made suggestions that may improve the study and its presentation and interpretation, many thanks for their efforts! I am very much looking forward to a reply by the authors, which I expect will be a very valuable discussion of these points. I do not think that every suggestion must be followed, but a few of them are certainly worth a trial (e.g. the smplification of haplotypes). I leave it to the authors to choose among those and argue how best to improve the manuscript, and I hope it will be convincing to us.

RESPONSE: we appreciate indeed the time and constructive suggestions of the Reviewers and Editor. We have addressed all of their comments, please find below itemized responses.

REVIEWER #1 COMMENTS

COMMENT: *Long-distance pollen dispersal indeed is a focus of several recent studies. However, solution how to estimate it are generally lacking. This manuscript provides one of the solutions that might be applied to conifers because the authors used paternally inherited markers (cpSSRs). Any attempt to bring us closer to solving the dilemma about the extent of long-distance pollen-mediated gene flow deserves attention. I generally find the manuscript well designed. The sample size and markers used are fine. Novel aspects include the Bayesian estimation method, supported by relevant simulations. The general conclusion supports our expectations that pollen-mediated gene flow in trees such as Scots pine might be extensive and may significantly contribute to newly generated offspring cohorts. I fully support this manuscript for publication in PLOS ONE. However, I have some comments and suggestions that might appear useful for the authors to improve the manuscript.*

RESPONSE: we appreciate the positive assessment and constructive comments.

COMMENT: *The authors mention in several places that haplotype frequency estimation uncertainty has little effect on the precision of the immigration levels. This problem is only briefly mentioned in methods that in one of the simulations, all populations have equifrequent haplotypes (line 301). The frequency distribution of haplotypes, especially when they are multiple, as in Scots pine, raises some questions about the sample size to approximate actual frequencies representing particular populations. With multiple haplotypes with very low frequency, the sampling variance for the frequencies of low-frequency haplotypes are large, and the ability to include a particular haplotype in the sample is small. This at least should be mentioned in the manuscript.*

RESPONSE: the reviewer is absolutely right; uncertainty about the frequency of rare haplotypes is an important issue in our system, one that had actually driven the design of our simulation scheme from the start. We apologize because we think we did not explain sufficiently well how we conducted the simulations. We did not use equifrequent haplotypes to simulate adult and

offspring samples. Instead, we actually constructed simulated haplotypic frequency distributions resembling the empirically observed ones, but allowing for much larger numbers of undetected rare haplotypes. In order to set the assumed haplotypic frequencies during the simulations, we did estimate them based on observed counts under the *prior* assumptions of equal global frequency over all populations and that their actual number may be up to 4x larger than the observed one; but note that this prior was updated based on observed counts, so that the posterior values used to simulate samples are not equifrequent but rather show an unevenness similar to the empirical one (see Rannala & Mountain 1997). This is a way to acknowledge a priori that there may be many more haplotypes than we actually observed and that many of them will remain undetected in the small samples used to infer both haplotypic frequencies and migration rates.

In the simulations, we incorporate the uncertainty in the estimation of rare haplotypes in two ways: (i) assuming that there were up to 4 times more haplotypes in the populations that we actually observed in our field samples; and (ii) once the assumed population haplotypic frequencies had been used to simulate the (small) adult and offspring samples, we subsequently assume the frequencies to be unknown during the inference step, estimating them based on the small simulated adult and offspring samples, just as in reality. In this way, our simulation scheme incorporate estimation errors owing to small adult and offspring samples, including those due to uncertainty in the estimation of the frequency of rare haplotypes, many of which actually remain undetected in the simulated samples.

We have tried to clarify the rationale and the implementation of these ideas in the manuscript, both in the Supplementary Appendix S3 (L34-39) and in the main text (L323-326).

COMMENT: *A variance of cpSSR haplotypes also depends on the variance of the fecundity of adults in the 'source' populations. I wonder if this model is sensitive to the variation of haplotype frequency due to the variable male fecundities in the source populations?*

RESPONSE: this is a good question to which we devoted a fair amount of thinking early on. Individual fecundity variation certainly has an effect on several models to estimate dispersal, mostly those based on parentage assignment, where it may determine parentage likelihoods as much as spatial distance factors. However, in our different statistical exercise of genetic assignment to populations, it should be expected to have a minimal effect. The reason being that our eqn. 1 is simply assuming that individual pollen fecundity is independent of the individuals' haplotype, whereas it is *NOT* assuming that all individual pollen sources have the same fecundity. Given that the number of pollen donors contributing to the population pollen cloud can be safely assumed to be very large, there is no reason to expect an unequal distribution of fecundities across paternal haplotypes (even if it is unequal across individual trees). We have added a clarification before eqn 1 to indicate that we are assuming haplotype-independent pollen fecundity (L246).

COMMENT: *What is the age of plantations? Did they reach the full capacity of pollen production since establishment?*

RESPONSE: they were introduced early in the second half of the 20-th century (L171), so they are approximately 60-70 years old and have probably reached full reproductive competence. We have added a short clarification on this regard (L172-173).

COMMENT: *What is the approximate proportion of native populations and plantations. Based on Fig 1, plantations demonstrate a low proportion of forested area as compared to natural populations.*

RESPONSE: the proportions within the study area are approximately 1/4 and 3/4 for planted and native stands, respectively. There are however larger planted stands further away, up to 30-40 km west and south of the study area. We have included this additional information in L516-517, acknowledging that they could represent a potential unsampled source of immigrant gametes (and mention that, if they were truly contributing, it would only tend to increase the inferred scale of among-population gene flow in our system) (L518-520). See also reply to the related comment below.

COMMENT: *The origin of plantations might be of some importance. Their relatedness to one or both natural stands may cause an overestimation of immigration levels for plantations. If those plantations originate from seeds derived from natural local stands, then the mixture of both natural stands may likely form a likely source of populations. This is probably not a very sensible idea, but I would try to generate an artificial (in silico) population generated as a random mixture of haplotypes from the two natural stands, then mimic this mixture population as a source of pollen (instead of actual plantations), and see how this could influence estimation process.*

RESPONSE: unfortunately, the origin of the plantations is unknown, because at the time it was not common practice to document forest reproductive material origin. Our guess (L170) is that they are of local origin, but this is just a hypothesis. We also ignore from how many mothers were the seeds collected from, it was not uncommon at the time to do it from a rather limited number of them. Overall, although the suggested approach has some theoretical appeal, we believe there is too much uncertainty on the origin and on the characteristics of the seed collection for us to recreate it realistically. In fact, it would be very unlikely that we could design a simulation scheme more realistic than our current approach, where we are using the empirically observed haplotypic frequencies of the plantations to construct assumed haplotypic frequency distributions for the simulations, which are conditioned on the observed counts but also allow for the presence of higher numbers of low-frequency undetected haplotypes.

COMMENT: *Line 174, based on fig 1, it cannot be said that sampling of adults and offspring was random. It was not random but done in kind of lines/transects/trails ???) both in natural stands and plantations. It could be random in respect to haplotypes, because I have never seen any fine-scale spatial genetic structure in any conifer based on cpSSRs.*

RESPONSE: correct, we have rephrased to indicate that adult and offspring were sampled along transects (L183, L191).

COMMENT: *Line 177, the distance between sampled adults may not be so essential to avoid relatedness based on cpDNA markers in conifers.*

RESPONSE: it is true, it is rather unlikely to find nearby “closely-related individuals” in terms of their cpDNA, and this was a rather conservative measure in that regard. In fact, the separation distance was also meant to spread samples for better haplotype population frequency estimation. We have rephrase to accommodate these two ideas (L187).

COMMENT: *Are there any additional scots pine stands in the area not covered by figure 1, that could be the source of immigrant pollen? This is important for discussion.*

RESPONSE: yes, this is a good point. Although we sampled a substantial proportion of the distribution area of the two studied native populations and the neighboring plantations, distant scattered planted stands actually extend beyond the study area, mostly up to 30-40 km to the West and South. We now mention this in the Discussion (L516-517), in the paragraph where we

already acknowledged the possibility of pollen immigration from unsampled external sources (which would only tend to increase the inferred scale of among-population gene flow in the study system). See also the response to a related comment above.

COMMENT: *Simulations were fine, but they all assumed that the frequency distribution of haplotype frequency was precisely estimated. I wonder, what would be the effect if the frequency of haplotypes would be estimated with bias?*

RESPONSE: we apologize again for not being clear on this matter. As replied in more detail in our response to the related comment above, we did not assume when testing the model that the adult haplotypic frequency distribution was known (nor that it was precisely estimated). As described above, we certainly assumed initially a haplotypic frequency distribution (conditioned on observed haplotypic counts and allowing for much higher number of low-frequency undetected haplotypes) in order to simulate adult and offspring samples of the same size as the empirical ones. But once the samples were simulated, we then assumed that the haplotypic frequencies were unknown, and "forced" the model to estimate them from the small adult and offspring samples, in the same way as during inference from real data. In this way the simulations reflected errors associated with haplotypic frequency estimation. As also mentioned above, we have added some clarifications on this matter (Supplementary Appendix S3 L34-39 and main text L323-326).

COMMENT: *Table 3 Suggest putting '-' instead of 0.000 on the diagonal.*

RESPONSE: done

COMMENT: *Line 482, the statement 'we found a large proportion of recruits sired by non-local males' does not seem appropriate because this sounds like the evidence-based on paternity analysis (genetic exclusion). I suggest talking about this in terms of 'probability' of non-local siring.*

RESPONSE: rephrased to "the estimated frequency of recruits sired by non-local males was high" (L502-503).

COMMENT: *Line 491, Scots pine is known to be protogynous at individual and population levels, which means that female flowers are exposed to pollen before local pollen shedding, contributing to increased pollen immigration. There is a vast amount of references that document this phenomenon.*

RESPONSE: right, it was inadequate to say that we had no prior reason to expect such phenological pattern. We have rephrased and added a classical study on Scots pine pollination biology showing it (L509-512).

COMMENT: *Several important papers touch on the problem of extensive pollen-mediated gene flow in Scots pine and conifers and might be considered as references. Some of long distance pollen dispersal has been presented in:*
Lindgren, D., Paule, L., Xihuan, S., Yazdani, R., Segerström, U., Wallin, J.-E., & Lejdebro, M. L. (1995). Can viable pollen carry Scots pine genes over long distances? Grana, 34(1), 64-69.
Nilsson, J. E. (1995). Genetic variation in the natural pollen cloud of Pinus sylvestris: a study based on progeny testing. Scandinavian Journal of Forest Research, 10(1-4), 140-148.
Burczyk, J., Sandurska, E., Lewandowski, A,. (2019). Patterns of Effective Pollen Dispersal in Larch: Linking Levels of Background Pollination with Pollen Dispersal Kernels. Forests, 10(12), 1139.

RESPONSE: thanks for these relevant suggestions; we have added them to the manuscript.

COMMENT: *Line 519, wrong citation, the cited paper referred to the problem of gene flow between species in terms of hybridization and should not be confused with gene flow among populations of the same species. Try to find a better reference. Indeed, in the case of low to none genetic differentiation among populations measuring the levels of contemporary gene flow based on differentiation is inefficient.*

RESPONSE: true, the cited paper referred to the specific case of hybridization inference, even if it illustrated the same kind of methodological problem that we were referring to (the difficulty to distinguish shared ancestral polymorphism from gene exchange among population or taxa). We have replaced the paper with a more relevant general review on the topic (Marko & Hart 2011).

COMMENT: *Line 527-530, it is not only gene flow that determine differentiation but also (rather) the intensity of selection of adaptive traits. The intensity od selection in trees is high, because of massive production of offspring (in climax conditions on average one adult is replaced by one offspring, but each individual may produce thousands to millions of offspring over a lifetime).*

RESPONSE: we fully agree, we were not suggesting that gene flow would preclude adaptive differentiation, but just that it would have tended to counteract it, producing allele frequency changes in opposite direction than divergent selection among populations. The next paragraphs of the Discussion elaborate substantially on these topics, including the hypothesis that the intensity of divergent selection has been high enough to enable local adaptive divergence despite high gene flow (L562-565 and L571-574).

COMMENT: *Line 613-615, but there are methods to deal with genotyping errors and nulls in nuclear markers, see Chybicki 2018 (Chybicki, I. J. (2018). NMpi-improved re-implementation of NM+, a software for estimating gene dispersal and mating patterns. Mol Ecol Resour, 18(1), 159-168. doi:10.1111/1755-0998.12710)*

RESPONSE: yes, we are aware of that elegant and advanced method, which is specific to parentage assignment and thus not suitable for our data. We were implicitly referring to the different statistical exercise of genetic assignment of individuals to populations, for which there is actually also a method to deal with genotyping errors and null alleles when using nuclear markers (we cite it in the text). We have added a clarification to indicate that we are referring to genetic assignment methods (L638-640).

COMMENT: *Line 617, where the effect of step-wise length scoring errors simulations results are presented? Cannot find it.*

RESPONSE: sorry for the confusion, we chose not to include them, nor the full model that jointly estimates mistyping rates. The reason being, as we mention in the text, that because of the high number of low-frequency haplotypes: (i) migration estimates were identical for scenarios with and without mistyping (i.e. mistyping had no effect on migration estimates obtained with the model ignoring mistyping); and (ii) therefore it was neither meaningful nor possible to jointly estimates mistyping rates accurately; trying to jointly estimate them simply results in higher variance of migration estimates and strong underestimation of mistyping rates. We now made clearer that these results are not shown, as they can be summarized in a couple of sentences.

COMMENT: *Lines 631-642, some of the future perspectives of research cover issues not related directly to the studied phenomenon – gene flow, but focus on the studying of patterns and evidences of genetic local adaptation.*

RESPONSE: we have now removed the last lines about local adaptation experiments (L663-668).

REVIEWER #2 COMMENTS

COMMENT: *The manuscript is focused on the estimation of pollen-mediated gene flow in a wind-pollinated conifer (Pinus sylvestris) at the southern margin of its distribution range in Spain. However, the focus is divided into two components: the biological questions related to long-distance pollen dispersal and the methodological improvements made on earlier statistical methods developed by some of the authors. The range of long-distance gene dispersal remains a largely unanswered question, mostly due to how difficult such dispersal events are detected. In wind-pollinated trees, pollen-mediated gene flow is mostly inferred using paternity exclusion which is ineffective in determining distances outside the local population. The methods used in this study rely on the opposite approach, where pollen gametes are fractionally assigned to different, discretely defined gene pools, each of which representing a different geographic location. Such an approach is quite well known and long-used by the community and resembles ideas implemented in the BayesAss software, but adjusted specifically to plants.*

RESPONSE: yes, we believe that the potential of well-known genetic assignment methods (e.g. BayesAss and others) still needs to be fully applied and adjusted to plant dispersal studies.

COMMENT: *Regarding the biological merit, the study seems to be inspired by the phenotypic divergence between pine populations, which provides a relevant basis for the hypothesis about migration and, possibly, divergent selection. The latter is mentioned as a hypothetical factor determining differences in early growth and phenology between the two population types, especially if recurrent migration takes place in both directions.*

RESPONSE: yes, we hope the present study will provide useful demographic information to interpret the results of future experiments specifically testing local adaptive patterns of these study populations.

COMMENT: *Generally, and especially taking into account the criteria of the PLOS One, I believe the manuscript is suitable for publication. The hypothesis is clear and sound. The methods are correct. Even if the biological findings are not truly novel (Scots pine is long known for its high pollen dispersal potential), they offer some novelty, especially in the part related to methodological improvements. However, before the final decision, I recommend some revisions according to the following suggestions (provided point by point):*

RESPONSE: we appreciate the positive comments. It is certainly true that Scots pine is one of the archetypical examples of high pollen dispersal potential, although (as well as for any other tree species) there are few empirical confirmations of *effective* long-distance pollen dispersal, especially into large populations.

COMMENT: *L178: It is generally acknowledged that a presence of relatives in a sample influences estimates of parental (say ancestral) allele frequencies. Such "ancestral" frequencies are typically needed to estimate unbiased genetic indices related to the probability of identity by descent in the current generation. But, as long as I understand, the estimation method used in this study does not involve such indices, but it is focused on assigning next-generation (progeny) genotypes*

to the current-generation propagule sources. Such an approach relies on the current generation allele frequencies, and not the previous generation (ancestral) ones. Therefore, intentional avoiding relatives can introduce ascertainment bias to estimated gene pools of current seed and pollen sources. Given the species' dispersal potential, family structure is likely to be very low so the mentioned bias has likely a negligible impact on the presented results. On the other hand, and seeking generality, in the case of strong family structure, the bias may no longer be ignorable. In my opinion, a random sample from a source population should be used. I would like to know opinion of the authors on this issue.

RESPONSE: the reviewer is right, the used method does not require inferring ancestral frequencies; it rather relies on accurate estimation of the haplotypic frequencies in the pollen pool produced by current (present-day) populations. We believe that spreading the sampling will result in a similar sampling probability for every spatial family group (if any), and thus it will not bias the estimation of population haplotypic frequencies in presence of family structure (even if some/most families are only sampled once or even remain unsampled). On the other hand, for a given (necessarily limited) sample size, spreading samples allows increasing the spatial sampling scale, which may help capturing potential spatial genetic structure over larger scales due to genetic isolation by distance. In conclusion, although it is true that improperly sampling family structure may bias genetic relatedness indexes based on identity-by-descent probabilities, we do not expect our very different model to be affected by this issue. We believe our sampling scheme is producing approximately random samples of male gametes in the pollen cloud over the study area.

COMMENT: *L222: lower-case J should be used in "... from population J"*

RESPONSE: fixed (L233), thanks.

COMMENT: *L255: the use of "uninformative" for uniform priors is not generally accepted because such prior distributions still introduce some information. Please use "weakly informative" instead.*

RESPONSE: we have replaced the term "uninformative" with "flat" (L273), the most objective term we could think of, given that we are strictly using flat priors. We also explain what our prior means (L278-280), letting the reader decide on how "informative" it is (as indicated by the reviewer, we acknowledge there is a lot of debate on this terminological and statistical issue).

COMMENT: *L265: the simulations were run using the posterior haplotype frequencies (L299) obtained under (I guess) the assumption of the uniform priors. It is then not surprising that the other priors used to model simulated data gave worse results. By the way, concentration parameters (alphas) of 1/K or 1/J in the Dirichlet distribution represent the so-called Jeffreys prior which is considered to be an uninformative prior under some assumptions.*

RESPONSE: we have removed this comment from the manuscript to avoid confusion (L280-283). We believe that the flat prior that we had used (and have chosen to maintain) is more adequate than Jeffreys prior in our particular case, as the latter would be in fact assuming a priori that all haplotypes have the same frequency within populations, and we have plenty of prior evidence from many species and populations suggesting that this is very rarely the case (allele and haplotype frequency distributions are typically skewed within populations). Same goes for pairwise migration rates among populations. Our Dirichlet prior with alpha=1, by contrast, does not give higher weight to any allele frequency (or migration rate) distribution a priori, so in particular it does not assume that the uniform (equifrequent) distribution of haplotype frequencies or migration rates is more likely a priori.

Note, on the other hand, that we did initially assume equifrequent haplotypes when constructing the frequency distribution necessary for the simulations, but this was the prior distribution over all populations before updating with the observed counts within each population, so that in the end the simulated haplotypic frequency distribution within each population, used to simulate adult and offspring samples, was not uniform in any population, but skewed in a similar way as the empirical within-population distributions (while also allowing for larger numbers of haplotypes).

COMMENT: *L268: maybe you mean "Chain rule"*

RESPONSE: we refer to "Bayes' theorem" or "Bayes' rule" on the (unnormalized) posterior density. The terms "Bayes' rule" and "Bayes' theorem" are widely used interchangeably in the statistical literature (e.g. page 7 in Gelman et al "Bayesian Data Analysis" Chapman & Hall.)

COMMENT: *L278: but it does not reduce the ascertainment bias due to non-random sampling from source populations*

RESPONSE: see reply to related comment above

COMMENT: *L281: "than" should read "to"*

RESPONSE: fixed, thanks (L299).

COMMENT: *Table 3: Using a bolded font instead of italicized one should help "extracting" the significant value out.*

RESPONSE: changed.

COMMENT: *L456: Given so wide credible intervals for migration vectors, I am not convinced that gene flow is generally asymmetric. To conclude that, the fit (or probability) of the estimation model needs be compared vs. the null model of symmetric gene flow.*

RESPONSE: the reviewer is right; credibility intervals for the two mentioned estimates are virtually fully overlapping, so we have deleted the inadequate conclusion of asymmetry (L476).

COMMENT: *L459: remove double "that"*

RESPONSE: removed (L479), thanks.

COMMENT: *L520: but the potential can depend on the species absolute number, i.e. the number of trees shedding pollen. In the case of substantial demographic depression, when the number of trees per forest patch is of order of tens or less, the total amount of pollen grains can be insufficient to effectively pollinate female strobili in another patch (the effect of pollen dilution).*

RESPONSE: agreed, we have qualified our conclusion, referring to the case of fragmentation of relatively large stands (L542-545).

COMMENT: *L537: The estimation of pollen migration is based exclusively on chloroplast genetic data. In order to extrapolate the observed migration patterns to the nuclear genome, the assumption of cyto-nuclear equilibrium must hold that would imply equivalence of divergence rates (up to the ploidy factor) between cp and nuclear genomes. However, divergence rates can*

differ due to selection affecting differently the two genomes. Assuming that the phenotypic divergence observed in the study populations has a genetic basis, it is mostly related to the selection on the nuclear genes. Then, cp and nuclear genomes may not be equivalent in so-called effective (i.e. after selection) gene flow patterns. Under such circumstances, neutral cpDNA can over-estimate the effective gene flow. I would like to know opinion of the authors about that.

RESPONSE: we are not sure we fully understand this caveat. Our model is just providing an estimate of the frequency of contemporary F1 immigrants in the samples, meaning either seeds dispersed from present-day local mothers pollinated by present-day non-local fathers or seeds dispersed from present-day non-local mothers pollinated by present-day non-local fathers. We are certainly using cpDNA markers to obtain this estimate, but we are doing so to measure the contemporary proportion of F1 immigrants, without any assumption on cyto-nuclear equilibrium. It could well be that selection operating on these F1 immigrants and subsequent backcrosses resulted in some kind of cyto-nuclear disequilibrium in later generations, so that effective gene flow tends to be reduced (relative to F1 immigration) and unequally so for oganellar vs. nuclear DNA. However, that does not affect our inference or our conclusions. In fact, we precisely argue in the Discussion that the potential long-term reduction of effective gene flow due to selection could reconcile the high estimated levels of *contemporary* gene flow and strong adaptive population divergence (L574-577). Whether this reduction will affect differentially nuclear vs. organellar DNA is beyond our goals and discussion framework. And as said, this potential cyto-nuclear disequilibrium would in any case not affect our inference of contemporary immigration.

COMMENT: *L602: Rather than affecting "errors", large samples will increase precision of estimates. However, increased precision is only one side of the coin. If accuracy remains unaffected, higher precision will lead to false positives, such as significant (non-zero) migration in the case of no migration. This may limit the utility of the proposed method to non-zero migration cases only. Here, again, the lack of formal test of the null hypothesis (no migration) decreases the value of the proposed method.*

RESPONSE: we fully agree, a biased (i.e. inaccurate) estimator with lower variance can lead to more misleading inference than one with the same bias and higher variance (as some people tend to wrongly regard narrow confidence intervals as evidence of accuracy). That is precisely why we were, and are, specifically referring to the reduction of the bias (increase in accuracy), not of the variance (L626-627).

COMMENT: *S2 Appendix: L49-51: If 260,000 iterations are used in total, including 10,000 discarded iterations for burn-in, then the thinning of 25 samples gives 10,000 samples from the posterior, and not 1,000 (as said in the text).*

RESPONSE: fixed, thanks (yes, we did use 10,000).

REVIEWER #3 COMMENTS

COMMENT: *The manuscript addresses the important topic of effective long distance gene flow by pollen among tree populations. Accurate estimations of this long distance pollen immigration is quite challenging for large wind pollinated tree populations. The authors observed high and asymmetrical pollen immigration rates in two natural Pinus sylvestris stands and discussed how this could be explained despite adaptive differences observed in a common garden experiment. The main merit of the paper is the presented Bayesian approach to simultaneously estimate haplotype frequencies and immigration rates. The weak point- which has also been addressed*

by the authors- is the high variation at the chloroplast microsatellites that lead to an extreme high number of haplotypes (line 361 ff.). Even with the high sample sizes presented in the paper all estimates of haplotype frequencies remained imprecise. As a consequence, nearly all pairwise comparison of genetic differences in haplotype frequencies are statistically not significant (line 323 ff., table 3) and all estimates of pollen migration rates had very large credibility intervals (line 389).

I suggest to apply a "rarefaction" approach to check the robustness of the estimated pollen immigration rates. With combinations of less than the six microsatellites the number of haplotypes will decrease and the confidence intervals of the frequency estimations will all get narrower. All measures could be repeated with different numbers of combined microsatellites.

RESPONSE: the very high within-population genetic diversity of Scots pine, in particular in terms of chloroplast haplotypes, is indeed a tricky issue for the purpose of gene flow inference. On the one hand, higher molecular marker polymorphism tends generally to increase genetic discrimination power, which is good for gene flow estimation; but on the other hand, higher diversity makes harder accurate estimation of haplotypic frequencies, which is not good. We have faced this problem in several previous gene flow studies that we have conducted with this species, and the "rarefaction" approach suggested by the reviewer is certainly an appealing one that we had indeed tried in the past, with the very same hopes formulated by the reviewer. However, what we have found in the past is that removing cpSSR regions reduced the accuracy of among-population gene flow estimates based on genetic assignment of individuals to populations, the reason being that the variance reduction in haplotype frequency estimation gained in this way is of lesser importance than the loss of precision and accuracy of gene flow estimates resulting from the decreased discrimination power. Of course, one can think of scenarios where the proposed "rarefaction" should work, for instance if the candidate source populations were fixed for alternative variants at a single cpSSR region (or fixed for alternative haplotypes using a combination of a few cpSSR regions). Unfortunately, we have never observed these favorable situations for the Scots pine populations that we have studied, because among-population divergence is typically low. Nevertheless, in order to double-check whether our inference could be improved for the present data set with the suggested "rarefaction" approach, we have tested it once more as follows:

We repeated the numerical analysis of the expected errors in the estimation of gene flow rates with our model, but considering several reduced combinations of the six available cpSSRs. In particular, we considered six different subsets of 5 cpSSRs (obtained by removing one different cpSSR at a time), four different subsets of 4 cpSSRs (obtained by removing two different cpSSRs at a time), and two different subsets of 3 cpSSRs (obtained by removing three different cpSSRs at a time). These combinations resulted in the observed number of haplotypes ranging from a high of nh = 247 (with all six cpSSRs) to a minimum of nh = 25 (with one of the 3 cpSSR combinations). For each subset we computed the expected root mean square error (RMSE) of gene flow estimates under two different gene flow scenarios, one without migration (scenario A) and one with migration rates similar to the estimated ones (scenario L). We also calculated the expected coverage rate of credibility intervals around migration estimates, as this is a more meaningful measure of the goodness of uncertainty estimation than the mere width of credibility intervals (credibility intervals may be very narrow but include the true value very rarely, being thus wrong and misleading).

We present here the results of these analyses, showing the expected RMSE (root mean square error) of immigration rate estimates obtained for each reduced subset of cpSSR. Rather than indicating the particular combination of cpSSR used in each case, we indicate the observed

number of haplotypes (nh) obtained with each combination. In red are the results for the full data set with all six cpSSR. These are the results for scenario A (no migration):

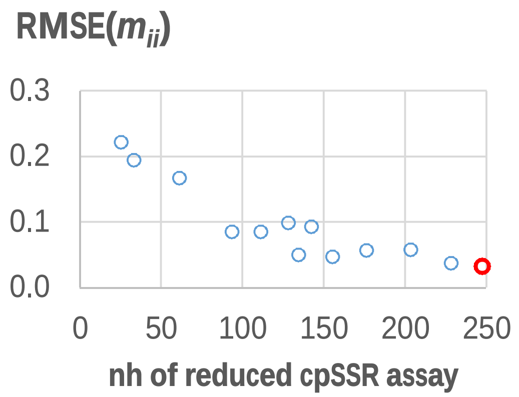

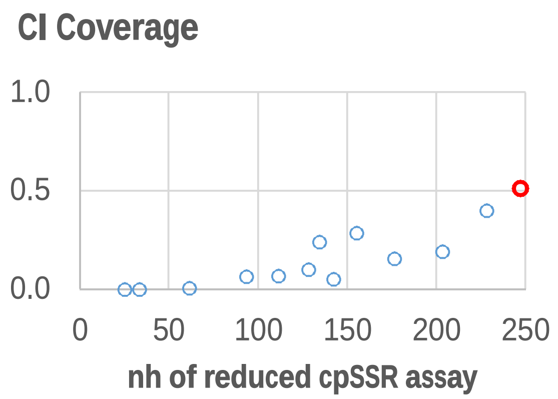

And the following are the results for scenario L (migration rates similar to estimated ones):

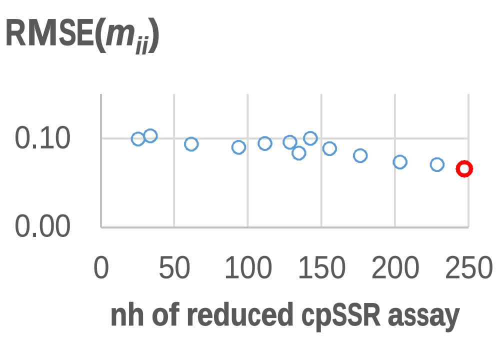

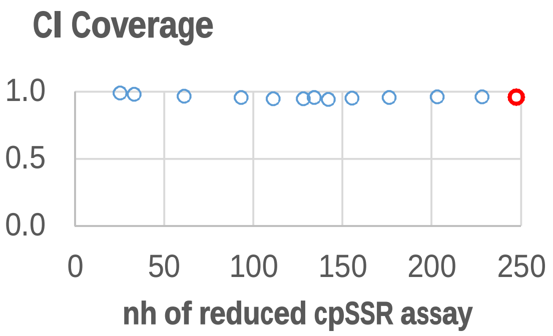

The graphs show that, for both gene flow scenarios, reducing the number of used cpSSR (and thus the number of haplotypes) invariably increases the expected error (RMSE) of migration rate estimates, with the minimum RMSE obtained with the full cpSSR assay. Results also show that reducing the number of used cpSSR either worsens the actual coverage of credibility intervals (for the first scenario) or virtually does not affect it (second scenario).

Overall, as we have previously found in our research, using reduced subsets of cpSSRs does increase the expected estimation error of gene flow rates among Scots pine populations, and it may also worsen the actual coverage of credibility intervals, making them less reliable (irrespective of their width). We have therefore chosen not to add new analyses with any reduced subset of cpSSRs in the manuscript itself.

COMMENT: *Line 180 ff.: Why offspring and not seeds have been used? How was the sampling design of the offspring?*

RESPONSE: we used 0-20 yr-old offspring because, given typically strong inter-annual variation in pollination and dispersal patterns, we considered more informative to obtain an average estimate of recent effective pollen gene flow (F1 immigrants) during the last couple of decades than one for a single season seed-crop. Offspring were sampled where found along the same

transects used for adult collection. We have added this missing information (L190-191 and L194-195).

COMMENT: *Line 482: Discuss the impact of the age of offspring: At least some of them might be older than a couple of years. And the haplotype frequencies in the adult population might have changed since then.*

RESPONSE: that is a fair concern, since we are assuming that our contemporary adult sample represents the paternal population of sampled (0-20 yr-old) offspring. However, we believe that any potential bias in haplotype frequency estimation due to this assumption should be negligible, because the populations are very large, with many thousands of individuals and because the stands are naturally regenerated only every 100-120 years (there weren't any important recent cuttings in the study area). The potential bias mentioned by the reviewer is probably far less important than the one simply associated with the usual samples sizes and spatial sampling coverage of this kind of studies.

We have added some clarifications to indicate that the range of sampled offspring ages translates into gene flow estimates that represent an average over approximately the last twenty years (L194-195). We now also acknowledge in the methods that we are implicitly assuming that haplotypic frequencies of contemporary adults are not significantly different from those of fathers producing effective male gametes during the last couple of decades, and why we think this is a reasonable assumption (L265-271).

COMMENT: *Line 537 ff.: One additional and simple explanation for the discrepancy of local adaptation and high effective pollen immigration would be that the pollen immigration has been overestimated. There are other population genetic and demographic parameters that could create differences between haplotype frequencies in adults and offspring of the same stand: e.g. drift effect due fertility variation of adults.*

RESPONSE: we now clarify that the referenced part of the discussion assumes that pollen immigration has not been overestimated (L560), and in a previous section of the discussion why we think it has not been (L520-523). On the other hand, as replied to a similar comment by the first reviewer, we do not think that male fecundity variation could be expected to introduce significant errors in our inference model, unless individual fecundity was dependent on the cpSSR haplotypes. Note that we are not saying that male fecundity variation does not exist, there is likely to be a lot, but if this variation is haplotype-independent (and there is no reason to expect it not to be independent) then fecundity variation should not induce much drift, because there is a very large number of pollen donors contributing to the pollen cloud.
