# Supplementary material for: Measuring recent effective gene flow among large populations in Pinus sylvestris: Local pollen shedding does not preclude substantial long-distance pollen immigration
Source: PLoS One. 2021 Aug 13;16(8):e0255776. doi: 10.1371/journal.pone.0255776 (PMC8362938; doi:10.1371/journal.pone.0255776)
Supplement: Attachment — Submitted filename: Review_07_05_21.docx [file pone.0255776.s006.docx]

Review of the manuscript:

Measuring recent effective gene flow among large populations in Pinus sylvestris: local pollen shedding does not preclude substantial long-distance pollen immigration

The manuscript addresses the important topic of effective long distance gene flow by pollen among tree populations. Accurate estimations of this long distance pollen immigration is quite challenging for large wind pollinated tree populations. The authors observed high and asymmetrical pollen immigration rates in two natural Pinus sylvestris stands and discussed how this could be explained despite adaptive differences observed in a common garden experiment.

The main merit of the paper is the presented Bayesian approach to simultaneously estimate haplotype frequencies and immigration rates. The weak point- which has also been addressed by the authors- is the high variation at the chloroplast microsatellites that lead to an extreme high number of haplotypes (line 361 ff.). Even with the high sample sizes presented in the paper all estimates of haplotype frequencies remained imprecise. As a consequence, nearly all pairwise comparison of genetic differences in haplotype frequencies are statistically not significant (line 323 ff., table 3) and all estimates of pollen migration rates had very large credibility intervals (line 389).

I suggest to apply a “rarefaction” approach to check the robustness of the estimated pollen immigration rates. With combinations of less than the six microsatellites the number of haplotypes will decrease and the confidence intervals of the frequency estimations will all get narrower. All measures could be repeated with different numbers of combined microsatellites.

Beside this main point I have only a few smaller remarks that might help to improve the paper:

Line 180 ff.: Why offspring and not seeds have been used? How was the sampling design of the offspring?

Line 482: Discuss the impact of the age of offspring: At least some of them might be older than a couple of years. And the haplotype frequencies in the adult population might have changed since then.

Line 537 ff.: One additional and simple explanation for the discrepancy of local adaptation and high effective pollen immigration would be that the pollen immigration has been overestimated. There are other population genetic and demographic parameters that could create differences between haplotype frequencies in adults and offspring of the same stand: e.g. drift effect due fertility variation of adults.
